# Use of Labelled tLyP-1 as a Novel Ligand Targeting the NRP Receptor to Image Glioma

**DOI:** 10.1371/journal.pone.0137676

**Published:** 2015-09-23

**Authors:** Hu-bing Wu, Zhen Wang, Quan-shi Wang, Yan-jian Han, Meng Wang, Wen-lan Zhou, Hong-sheng Li

**Affiliations:** NanFang PET Center, Nanfang Hospital, Southern Medical University, Guangzhou, China; University of Michigan School of Medicine, UNITED STATES

## Abstract

**Background:**

Neuropilin (NRP) receptors are overexpressed in glioma tumor tissue, and therefore may be a potential target for imaging markers. We investigated whether labelled tLyP-1, an NRP targeting peptide, could be used as the targeting ligand for developing reagents for imaging glioma tumors.

**Methods:**

The tLyP-1 peptide (CGNKRTR) was labeled with 5-carboxyfluorescein (FAM) or ^18^F-fluoride. A control peptide (MAQKTSH) was also labeled with FAM. The in vitro binding between FAM-tLyP-1 and U87MG cells and in vivo biodistribution of FAM-tLyP-1 in a U87MG glioblastoma xenograft model (nude mouse) were determined. The in vivo biodistribution of ^18^F-tLyP-1 was also determined by microPET/CT.

**Results:**

In vitro, FAM-tLyP-1 was strongly taken up by U87MG cells at very low concentrations (1μM). In vivo, FAM-tLyP-1 accumulated in glioma (U87MG) tumors, but uptake was minimal in the normal brain tissue 1 h after administration. The distribution of FAM-tLyP-1 in the tumor tissue was consistent with expression of NRP1. The tumor/brain fluorescence intensity ratio in mice treated with FAM-tLyP-1 was significantly higher than the control FAM-labeled peptide 1 h after administration (3.44 ± 0.83 vs. 1.32 ± 0.15; t = 5.547, *P* = 0.001). Uptake of FAM-tLyP-1 in glioma tumors could be blocked by administering an excess of non-conjugated tLyP-1 peptide. [Lys4] tLyP-1 was labeled with ^18^F to synthesis a PET (^18^F-tLyP-1). MicroPET/CT imaging showed the tumor was visualized clearly with a high tumor/brain radiolabel ratio at 60 min (2.69 ± 0.52) and 120 min (3.11±0.25).

**Conclusion:**

Taken together, our results suggest that tLyP-1 could be developed as a novel fluorescent or radio labelled tracer for imaging glioma.

## Introduction

Gliomas originate in the brain or spinal cord and account for approximately 30% of all brain and central nervous system tumors and 80% of all malignant brain tumors. High-grade gliomas are highly vascular tumors and have a tendency to spread [1–3]. They are not easily excised and often recur at the surgical margin. It is crucial that the glioma tumor is completely removed, while protecting surrounding normal brain tissue as much as possible, to improving patients’ survival and their postoperative quality of life. However, removing the tumor completely can be challenging because it is not easy to differentiate tumor from surrounding normal brain tissue before and during surgery [4–6].

Positron emission tomography (PET) and fluorescent imaging are highly sensitive imaging techniques that could be used to identify microinvasions from a glioma tumor into surrounding tissue to guide surgical removal of the tumor. There are several imaging reagents that are currently available including: ^18^F-fluorodeoxyglucose (^18^F-FDG), ^11^C-methionine, ^11^C-choline, and ^18^F-tyrosine for PET and 5-aminolevulinic acid for fluorescent imaging [7–15]. However, the available reagents are not tumor specific imaging agents and they are insufficiently sensitive and specific [7, 14–15]. Chlorotoxin (CTX), which can preferentially bind to matrix metalloproteinase-2 (MMP-2) in glioma cells, has been developed as an optical imaging contrast agent (CTX: Cy5.5) by conjugation to the fluorescent marker Cy5.5. CTX: Cy5.5 can potentially “paint” a glioma tumor and improve intraoperative detection and resection of malignant glioma [16–18]. However, CTX is a 36 amino acid peptide with four disulfide bridges bonds, and although it can effectively show glioma tumors, it would be difficult to commercialize due to its complex molecular structure [19–20]. Thus, there is an unmet need to develop a glioma specific, sensitive imaging reagent that can be easily commercialized.

The neuropilin receptors (NRP1 and NRP2) are single-pass transmembrane glycolproteins. NRP1 is the co-receptor for vascular endothelial growth factor (VEGF). NRP1 is over-expressed in angiogenic vessels in 98–100% of carcinomas, and plays a vascular-specific role in tumor-mediated angiogenesis [21–24]. Similar to NRP1, NRP2 can bind to members of the VEGF family of growth factors, including VEGF-A, VEGF-C, and VEGF-D, which are overexpressed in tumor lymphatic vessels and play an important role in modulating lymphatic metastasis [22–25]. The NRPs are also expressed in a wide variety of human tumor cell lines [22–29]. Many studies have reported that gliomas overexpress NRPs [29–34]. In addition, increased NRP expression positively correlates with glioma aggressiveness, disease severity, and poor prognosis [14, 31, 33]. Developing reagents to noninvasively visualize and quantify NRP expression levels will create new opportunities for imaging glioma, documenting tumor receptor expression, and more appropriately selecting patients to be considered for anti-NRP treatment.

The tLyP-1 peptide (CGNKRTR) is a truncated form of the cyclic tumor-homing peptide LyP-1 (CGNKRTRGC). tLyP-1 contains a CendR element and can penetrate tissue via the NRP1 and NRP2 dependent CendR internalization pathway. The studies by Roth L have shown that the tLyP-1 phage binds to immobilized NRP1 (120-fold) and NRP2 (8-fold) with markedly greater affinity than a control phage [35]. In addition, tLyP-1-conjugated nanoparticles selectively home to tumor tissue, penetrate the blood vessels, and enter the tumor parenchyma with greater potency than the parent peptide LyP-1 [35–38]. Given that NRPs are overexpressed in glioma tumor tissue and that tLyP-1 can enter tumors, we hypothesized that tLyP-1 might be a potential imaging ligand for glioma. To address this question, we conjugated tLyP-1 to the fluorescein of FAM and positron emitter, ^18^F-fluoride, respectively for use as a molecular imaging probe.

## Materials and Methods

### Materials

All of the commercially available chemicals were used as purchased. No-carrier-added ^18^F-F was obtained from an in-house PETtrace cyclotron (GE Healthcare, American). Reverse-phase extraction C18 Sep-Pak cartridges were obtained from Waters (Massachusetts, American) and were pretreated with anhydrous ethanol and deionized water immediately prior to use. The tLyP-1 peptide, FAM labeled tLyP-1 (FAM-tLyP-1), and control peptides (MAQKTSH) were custom manufactured by China Peptides Co., Ltd (Shanghai, China). The FAM label was attached to the amino group of 4th lysine in the tLyP-1 peptide and control peptide. The purities of the tLyP-1 peptide (99.65%), FAM-tLyP-1 (98.01%), and FAM labeled control peptide (98.4%) were determined by analytical high performance liquid chromatography (HPLC). Rabbit anti-NRP1 antibody was purchased from Abcam Ltd. (Hong Kong, China). Dylight 647-conjugated secondary antibody and 4,6-diamidino-2-phenylindole (DAPI) were purchased from Beyotime (Haimen, China).

Syringe filters and polyethersulfone membranes (pore size 0.22 μm; diameter 13 mm) were obtained from Millipore (Massachusetts, American). HPLC for ^18^F-SFB purification was performed using the Tracerlab FX_FN_ synthesis module (GE Healthcare, American) built-in HPLC system with a semi-preparative reverse-phase C-18 column (10×250 mm) and C-18 precolumn equipped with a UV detector and a radioactivity detector. The peptides were separated and identified using a Series L-10Ap HPLC system (Shimadzu, Japan) consisting of two LC-10ATvp pumps, a variable wavelength SPD-10ATvp UV detector, and a Flow-Count 3200 radio-HPLC Detector. The reverse-HPLC solvents were 0.1% CF_3_COOH in CH_3_CN (solvent A) and 0.1% CF_3_COOH in H_2_O (solvent B). HPLC purification was performed using mobile phase A 16% (0 min), 90% (25 min), 90% (35 min), and 16% (40 min) at a flow rate of 2.5 mL/min with a reverse phase column (Waters uBondapak^TM^ C18 column, 7.8×300 mm). Analysis was performed on a column (Waters XBridgeTM BEH130 C18 4.6×250 mm) using mobile phase A 16% (0 min), 95% (25 min), and 16% (30 min) at a flow rate of 1 mL/min.

### Tumor cell lines

A human glioma cell line U87MG that overexpresses NRP [31] was purchased from the Institute of Biochemistry and Cell Biology, Shanghai Institutes for Biological Sciences, Chinese Academy of Sciences (Shanghai, China). Cells were cultured in Dulbecco’s modified Eagle’s medium (DMEM) (Hyclone, America) supplemented with 10% fetal calf serum (Hyclone, America) at 37°C in a humidified 5% carbon dioxide-containing atmosphere.

### Animal model

Animal experiments were conducted under a protocol approved by the Nanfang hospital animal ethics committee at the Southern Medical University (Application No.: NFYY-2012-115).

Male and female BALB/C athymic nude mice (nude mice) 4–6 wk of age were obtained from the Laboratory Animal Center at the Southern Medical University. Glioma xenografts (U87MG cells) were inoculated into the mice by injecting 1 × 10^6^ cells intramuscularly into the left flank. Tumor xenografts were monitored until the largest tumor diameter was approximately 0.5–1 cm, which took 4–5 weeks.

### In vitro binding and blocking assay using FAM-tLyP-1 in U87MG cells

The ability of FAM-tLyP-1 to bind U87MG cells in vitro was assessed via fluorescence microscopy. U87MG cells were seeded on cover slips in 6-well plates and incubated in DMEM/F-12 (0.5 mL/well) overnight. The following day, the U87MG cells were incubated at 37°C for 1 h with different concentrations of FAM-tLyP-1 in PBS + 1% BSA (0, 1, 5, 10, 20, and 40μM). To assess whether FAM-tLyP-1 binding could be blocked by unlabeled tLyP-1 we incubated U87MG cells with FAM-tLyP-1 (1 or 4μM) in the presence or absence of tLyP-1 (20μM) for 37°C for 1 h. The cells were then washed three times with PBS. The slides were imaged by a blinded observer under blue light using a fluorescent inversion Olympus IX71 microscope (Olympus, America).

When imaging using the fluorescent microscope, we first used white light to confirm that tumor cells were in the field of view. Then, in the same field of view, we used the blue light to visualize the green light emitted from the cells and take the photos. The comparisons of FAM-tLyP-1 uptake by tumor cells in the in vitro binding and blocking experiments were strictly qualitative.

### The distribution of FAM-tLyP-1 in tumor tissue

U87MG tumor-bearing nude mice (U87MG tumor model) were intravenously injected with 150 μL of FAM-tLyP-1 (1mM). The mice were sacrificed after 1 h by cervical dislocation under ketamine/xylazine anesthesia. The tumors were removed, embedded in freezing liquid (McCormick, France), and sectioned with a CM1850 UVLeica freezing microtome (Leica, Germany) into 7 μm thick sections. The sections were mounted in DAPI-containing mounting media. NRP1 was stained using an anti-NRP1 antibody (1:1000) and a Dylight 647-conjugated secondary antibody. The tissue distribution of NRP1 and FAM-tLyP-1 peptides in the same tumor section was determined by a blinded observer using an Olympus DP71 fluorescent microscope (Olympus, America). Red fluorescence indicated NRP positivity and green fluorescence indicated positive staining with FAM-tLyP-1.

### Distribution of FAM-tLyP-1 by fluorescence techniques in the U87MG tumor models

Mice with U87MG tumor xenografts (n = 10) were randomly divided into an experimental group (n = 5) and control group (n = 5). The mice in the experimental group were intravenously injected with 150 μL of FAM-tLyP-1 (1mM), while the control group received 150 μL of FAM-labeled control peptide (1mM). The mice were sacrificed after 1 h by cervical dislocation under ketamine/xylazine anesthesia. The tumor and normal organs were then removed, washed with PBS at least three times, and then collected for imaging ex vivo. FAM-tLyP-1 uptake in the tumor and normal organs was described by a blinded observer under blue light with an exposure time of 60 s, using the Kodak in-Vivo Imaging System F (Kodak, American), and processed for fluorescence intensity analysis. The fluorescence intensity was analyzed visually based on the brightness of the green light indicating uptake of FAM-tLyP-1.

For whole animal imaging, U87MG tumor bearing mice were administered 150 μL of FAM-tLyP-1 (1mM) and then sacrificed and frozen at -80°C after 1.0 h. Each mouse was then cut into coronal sections. The uptake of FAM-tLyP-1 in the tumor and normal organs was observed under blue light using the Kodak in-Vivo Imaging System F (Kodak, American). To determine whether unlabeled tLyP-1 could block FAM-tLyP-1 labeling in the tumor, the mice were injected intravenously with 10 times the quantity of non-conjugated tLyP-1 peptide 30 min prior to FAM-tLyP-1 administration.

The intensity of FAM-tLyP-1 uptake in the tumor and normal organs was analyzed blindly using the Kodak MI analysis software (Kodak, American). The fluorescence intensity was analyzed visually from high to low levels based on a color scale of white, red, yellow, green, blue, and black. Regions of interest (ROIs) were drawn around the border of the tumor and normal organs on the fluorescence images and the average fluorescence intensity was measured. The tumor/non-tumor ratios (T/NT ratios) were calculated by dividing the fluorescence intensity in the tumor by that of the normal organs.

### Synthesis of PET molecular probe, ^18^F-tLyP-1

As previously described [32–34], [Lys4] tLyP-1 was labeled with ^18^F by coupling the Lys^4^ amino group with-succinimidyl-4-^18^F-fluorobenzoate (^18^F-SFB) under slightly basic condition (pH 8.5). ^18^F-SFB was synthesized, as previously reported and purified by HPLC [39]. The purified ^18^F-SFB was trapped on the cartridge and eluted with diethyl ether (5 mL) into a 10 mL V-vial in a customized module. The diethyl ether (Et_2_O) was removed under helium stream at ambient temperature and the dried labeling agent was reconstituted with the peptide in anhydrous DMSO. The conjugation between ^18^F-SFB and peptide was executed using 250 μg tLyP-1 peptide with DIPEA (40 μL) at 40°C for 20 min and was quenched by adding 5% acetic acid. The crude product was purified using semi-preparative HPLC. The ^18^F-tLyP-1 fraction was collected. The solvent was removed by rotary evaporation and the residue was resolubilized in saline. The formulated saline mixture was sterile-filtered into a sterile product vial.

### MicroPET/CT imaging analysis

MicroPET/CT scan was performed on a SIEMENS Inveon scanner (Siemens, Germany). U87MG tumor bearing mice (n = 5) were intravenously injected with 3.7 MBq (200 μCi) of ^18^F-tLyP-1. MicroPET/CT images were acquired as 10-min static images at 30, 60, and 120 min after the injection with the mice under isoflurane anesthesia. The images were reconstructed by a 3-dimensional ordered subsets expectation maximum (OSEM) algorithm and CT correction was applied for attenuation correction.

In the PET images, ROIs were measured with the Inveon Research Workplace (IRW) 3.0 software (Siemens, Germany). The ROI was determined by manually superimposing the ellipsoid volume of interest (VOI) to the target tissue. The activity concentrations were determined by the mean pixel intensity within each VOI, and converted to μCi/mL using a calibration constant. Assuming the tissue density of 1 g/mL, the ROI activity was converted to μCi/g and normalized as percent injected dose per gram (%ID/g). The tumor/normal brain ratios were calculated by dividing the ROI activity in tumor by that in the normal brain [40].

### Statistical analysis

Descriptive data were expressed as the mean ± standard deviation. Statistical Package for the Social Sciences, version 13.0 (SPSS Inc.), was used for the statistical analysis. The nonparametric one-sample kolmogorov-smirnov test was applied to assess for normality. A *P* value greater than 0.05 indicated the data was normally distributed. The T/NT ratios of FAM-tLyP-1 and FAM-control peptide were compared using the independent samples t test. One-way ANOVA was used to copare the uptake of ^18^F-tLyP-1 in the tumor and normal organs at 60 and 120 min post injection. A *P* value less than 0.05 was considered statistically significant.

## Results

### FMA-tLyP-1 is strongly taken up by U87MG cells

**Fig 1:**
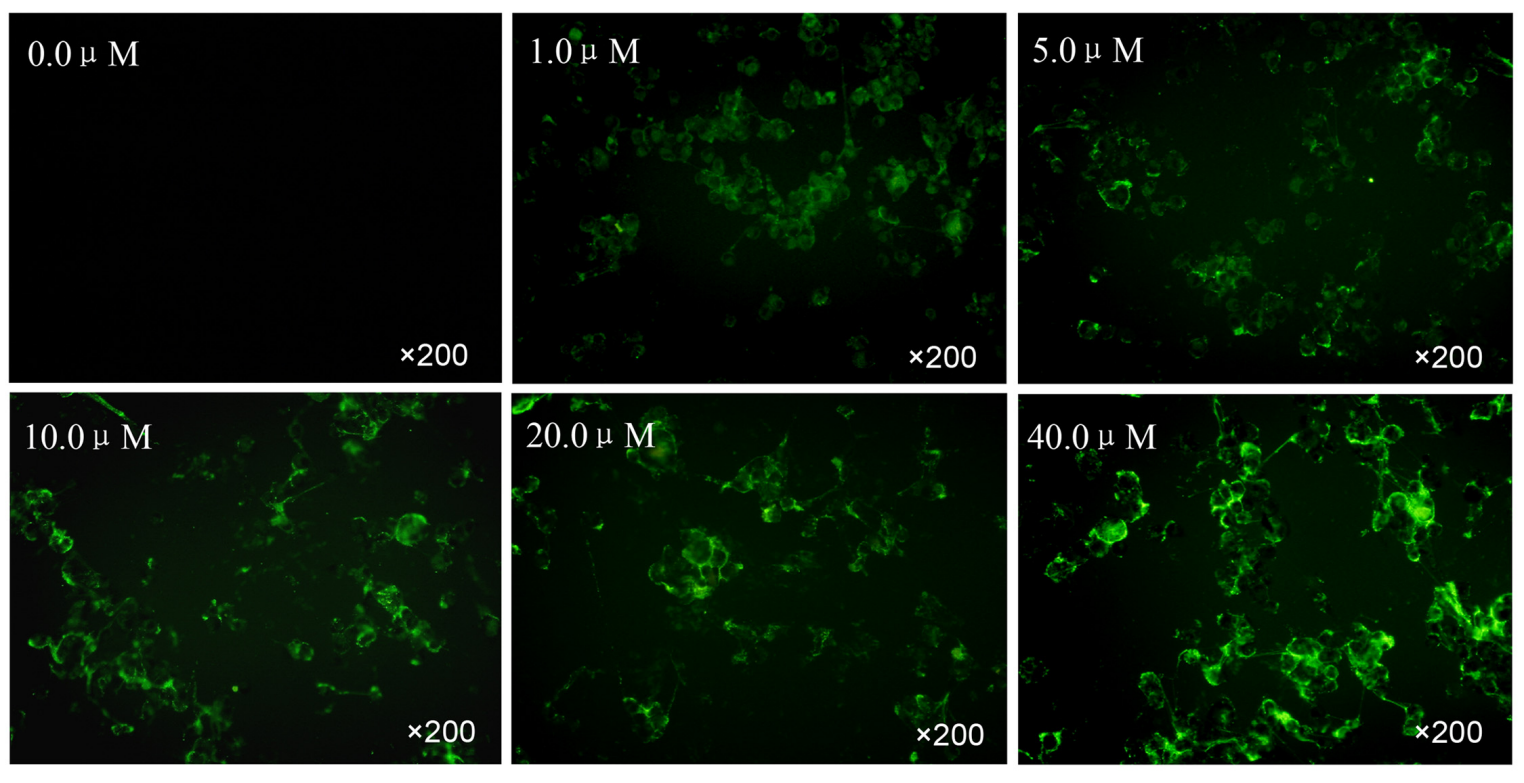
FAM-tLyP-1 uptake in U87MG cells in vitro. U87MG cells were incubated with different concentrations of FAM-tLyP-1 (0.0, 1.0, 5.0, 10.0, 20.0, and 40.0μM). The uptake of FAM-tLyP-1 in U87MG cells increases slightly in a dose dependent manner. FAM-tLyP-1: green; original magnification: ×200.

**Fig 2:**
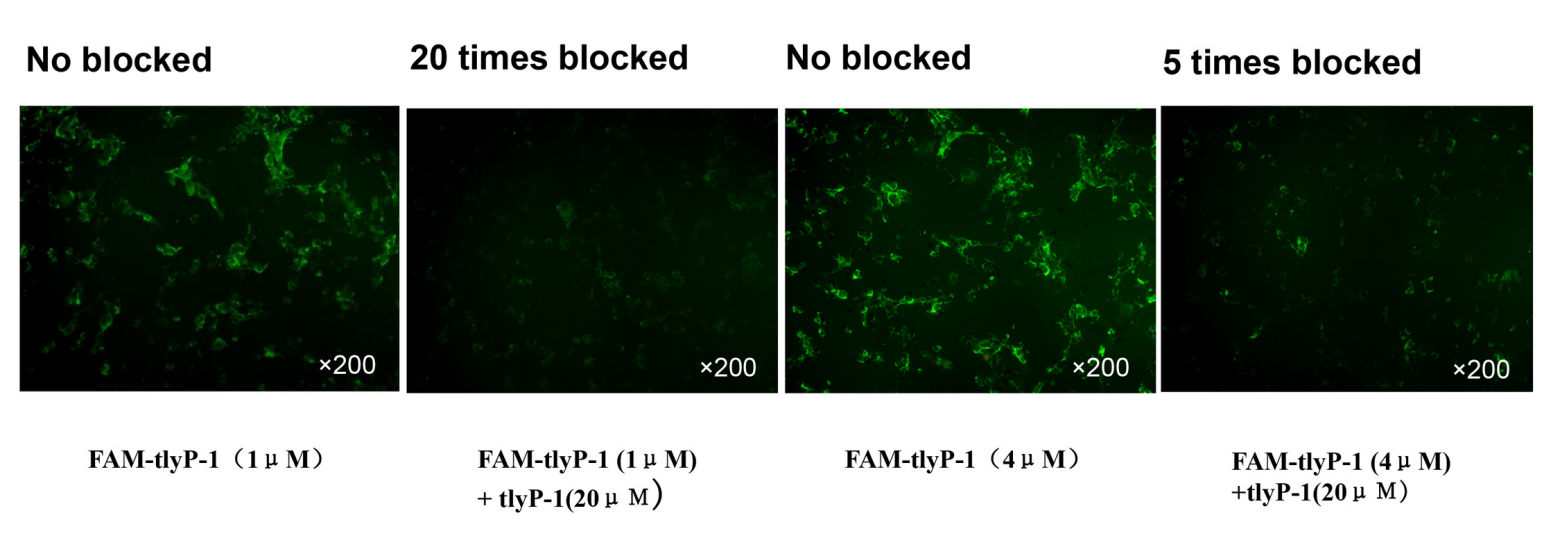
Blocking FAM-tLyP-1 uptake with non-conjugated tLyP-1. The uptake of FAM-tLyP-1 in U87MG cells is dramatically inhibited by incubation with an excessive quantity of non-conjugated tLyP-1 peptide. FAM-tLyP-1: green; original magnification: ×200.

We first tested the ability of FAM-tLyP-1 to bind to U87MG cells in vitro. FAM-tLyP-1 was strongly taken up by the U87MG cells at concentrations as low as 1μM. The uptake of FAM-tLyP-1 in U87MG cells increased slightly in a dose dependent manner (Fig 1). There was no apparent green fluorescence in cells treated with PBS. We then assessed whether unlabeled tLyP-1 could prevent the binding of FAM-tLyP-1. FAM-tLyP-1 uptake in U87MG cells was markedly reduced when the U87MG cells were first incubated with a 5- or 20-fold excess of non-conjugated tLyP-1 peptide. This suggested that tLyP-1 was likely competitively inhibiting FAM-tLyP-1 binding (Fig 2).

### FAM-tLyP-1 accumulates in tumor tissues that express NRP1

**Fig 3:**
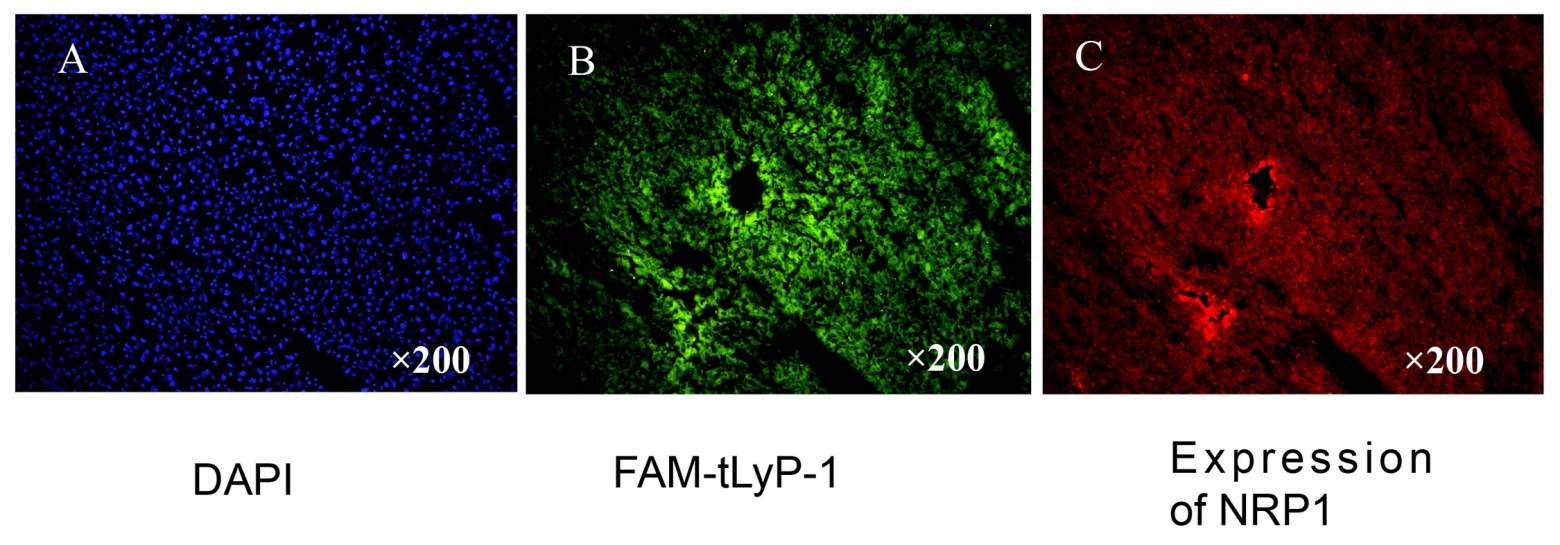
Representative images of FAM-tLyP-1 uptake in tumor tissue after 1 h in the U87MG tumor model. (A) The nuclei of tumor cells were visualized by DAPI staining. (B) Frozen sections under a fluorescent microscope. FAM-tLyP-1: green; (C) The same frozen sections as in B stained with anti-NRP-specific antibody. Original magnification: ×200; scale bars: 100μm.

We next assessed whether FAM-tLy-P-1 accumulated in tumors in vivo and whether its distribution was consistent with NRP binding in vivo. NRP1 expression was confirmed on the U87MG tumor tissue used in the present study (Fig 3C). In addition, FAM-tLyP-1 was shown to accumulate in the tumor tissue 1.0 h after administration by fluorescence microscopy (Fig 3B). The distribution of FAM-tLyP-1 was consistent with NRP1 expression in the same tumor section (Fig 3).

### FAM-tLyP-1 targets to the glioma tumor in vivo with a high tumor/brain ratio

**Fig 4:**
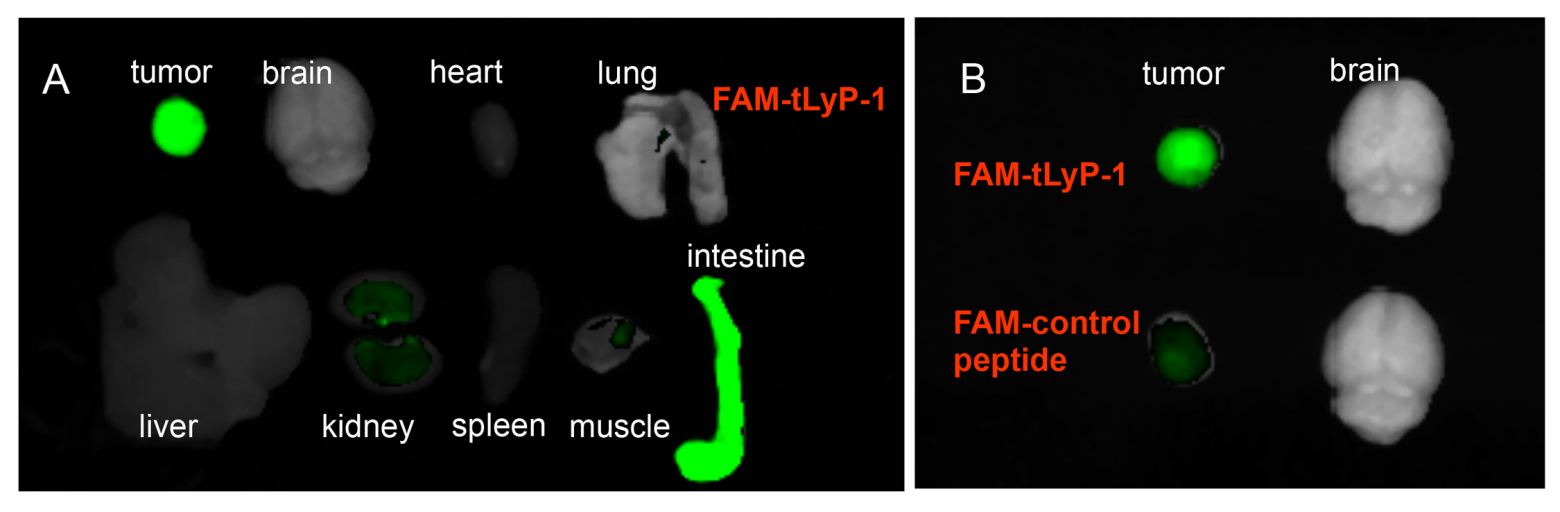
Uptake of FAM-tLyP-1 in tumor and normal organ tissues. (A) Tumor and normal organ tissues were removed and examined for fluorescence 1 h after FAM-tLyP-1 was intravenously injected into U87MG tumor bearing mice. (B) Uptake of FAM-tLyP-1 and FAM-control peptide in tumor and normal brain tissue.

To assess whether FAM-tLyP-1 had a sufficient signal to background ratio in vivo, we assessed the intensity of the green fluorescence in tumor tissue and normal tissue from individual organs in nude mice with U87MG tumors. One (1) hour after administration of the FAM-tLyP-1 the fluorescence intensity of FAM-tLyP-1 in the tumor was very high, compared to minimal fluorescence in the normal brain tissue (Fig 4A). The fluorescence intensity in the tumor was significantly greater than the normal brain with a tumor/brain ratio of 3.44 ± 0.83 (Table 1). The T/NT fluorescence ratio of the FAM-control peptide was 1.32 ± 0.15 in the brain after 1 h (Fig 4B), and was significantly lower than FAM-tLyP-1 (t = -5.547, *P* = 0.001; Table 1).

**Table 1:** Comparing the tumor/non-tumor (T/NT) ratios of FAM-tLyP-1 and FAM-control peptide in the subcutaneous U87MG glioblastoma xenograft model.

T/NT	FAM-tLyP-1	FAM-control peptide	t	P
T/brain	3.44±0.83	1.32±0.15	5.547	0.001
T/heart	4.61±1.00	1.94±0.26	5.727	0.000
T/lung	3.29±0.79	1.40±0.10	5.284	0.006
T/liver	2.34±0.36	1.28±0.25	5.315	0.001
T/kidney	0.88±0.74	0.57±0.51	0.784	0.456
T/intestine	0.84±0.35	0.87±0.30	-0.164	0.874
T/spleen	4.18±0.56	1.75±0.17	9.119	0.000
T/muscle	2.87±0.45	1.31±0.14	7.294	0.000

Note: Experiments were conducted in 5 mice. The data presented are the mean ± standard deviation.

The fluorescence intensity in other organs, such as the heart, lungs, spleen, and muscle was also minimal and the mean T/NT ratios in these organs also exceeded 2.0 (Table 1). The T/NT ratio was below 1.0 in two tissues of the intestine (0.88±0.74) and kidney (0.84±0.35; Fig 4A; Table 1).

**Fig 5:**
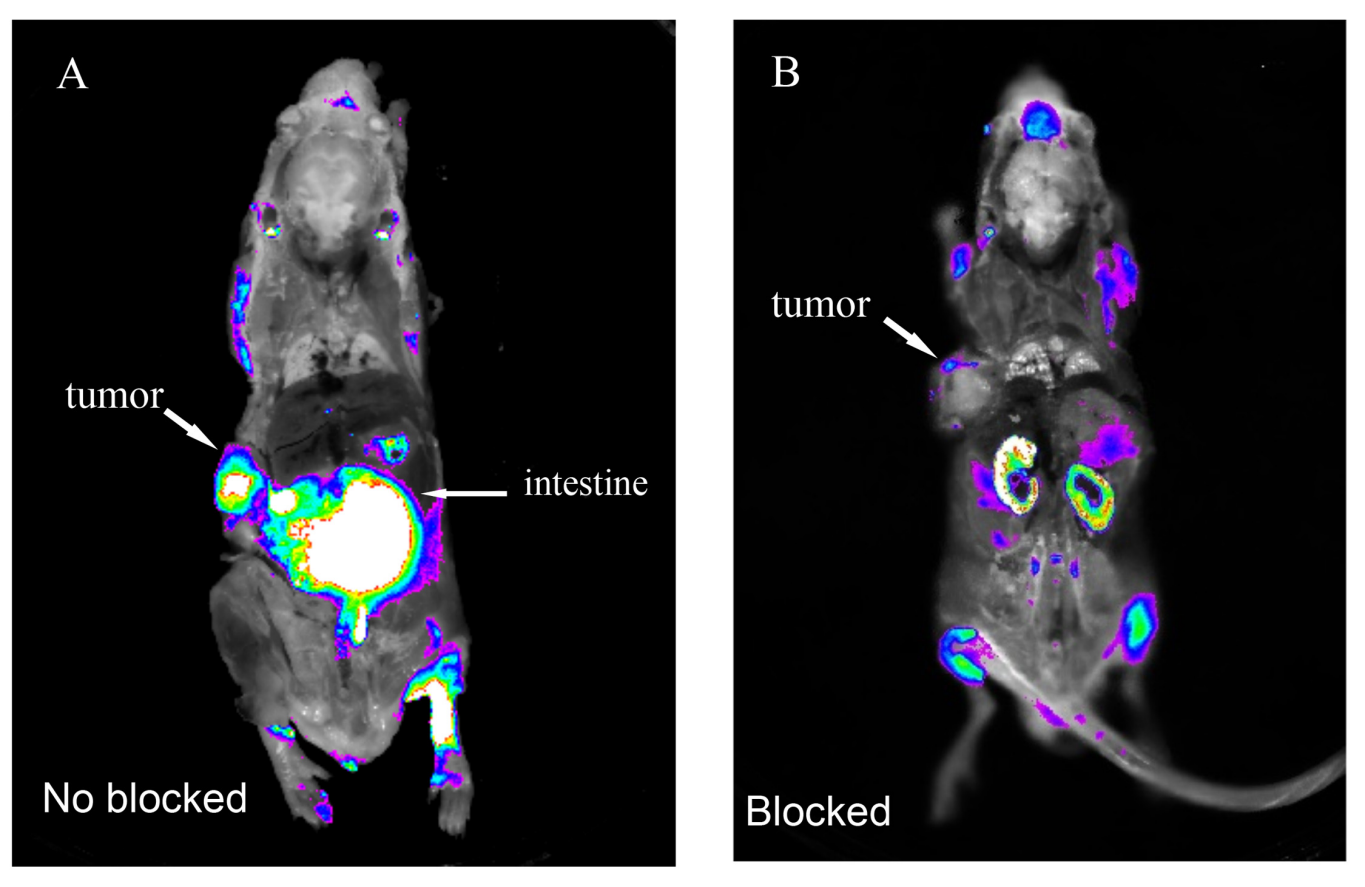
Uptake of FAM-tLyP-1 can be inhibited in vivo by tLyP-1 in the U87MG tumor model. (A) In vivo fluorescent imaging showing intense uptake of FAM-tLyP-1 in tumor tissue without inhibition by tLyP-1. (B) Minimal uptake of FAM-tLyP-1 in tumor tissue following administration of a 10-fold excess of non-conjugated tLyP-1 peptide. Images taken 1 h after administration of FAM-tLyP-1 by intravenous injection.

We also conducted whole animal imaging using coronally sectioned U87MG tumor bearing mice. These images also indicated high fluorescence intensity in the tumor, and minimal fluorescence in the brain, heart, lungs, liver, muscle, and bone. As with the isolated organs, intense fluorescence distribution was also noted in the intestine (Fig 5A). FAM-tLyP-1 uptake by tumors in vivo could be blocked by administering an excess of non-conjugated tLyP-1 peptide (Fig 5B). Overall, the results of the isolated organs and sectioned whole animal imaging were consistent, and indicated high levels of uptake by tumor, but not normal tissue.

### ^18^F-tLyP-1 micro-PET/CT visualizes the glioma tumor clearly

Having established that the fluorescent marker FAM-tLyP-1 was an effective imaging agent in vivo, we synthesized ^18^F-tLyP-1 for use in PET/CT imaging and assessed its in vivo imaging potential. The amount of time required for ^18^F-tLyP-1 synthesis was approximately 250 min, including the final HPLC purification. The overall radiochemical yield (without decay correction) was approximately 8–12%. The radiochemical purity of the labeled peptides was greater than 98% measured by analytical HPLC.

**Fig 6:**
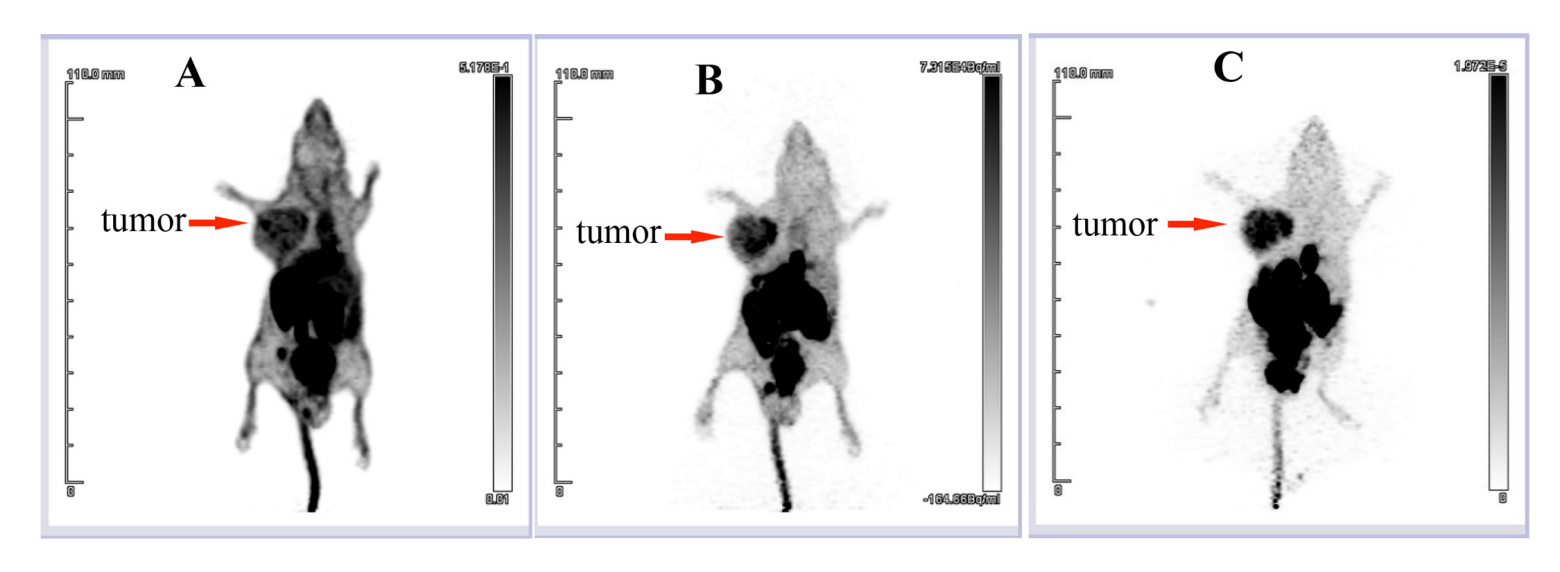
In vivo detection of glioma tumors using ^18^F-tLyP-1. In vivo microPET/CT MIP images in the U87MG tumor model at 30 min (A), 60 min (B) and 120 min (C) after injection of ^18^F-tLyP-1. The uptake of ^18^F-tLyP-1 in the tumor is intense, while minimal uptake is seen in the brain. MIP: maximum-intensity image.

**Fig 7:**
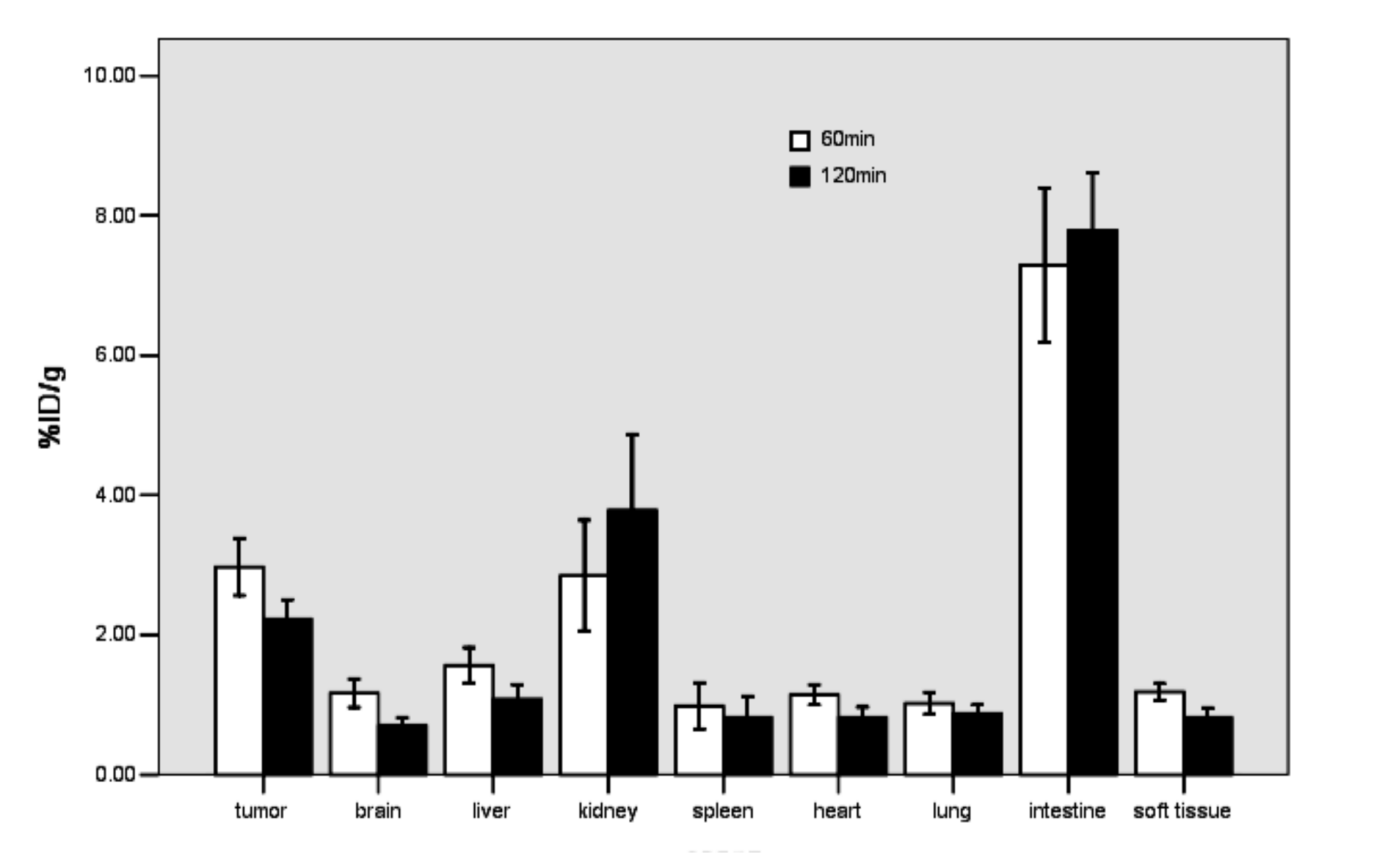
Biodistribution of ^18^F-tLyP-1 in U87MG tumor models. The biodistribution of ^18^F-tLyP-1 is shown at 60 min and 120 min after injection (n = 5 mice).

The ^18^F-tLyP-1 molecular marker was then tested in the U87MG tumor model. The maximum-intensity images (MIP) of the in vivo microPET/CT imaging from 30, 60, and 120 min after ^18^F-tLyP-1 injection are presented in Fig 6. There was high radioactivity in the tumor at 30, 60, and 120 min post administration. The radioactivities of the glioma tumors were 2.97 ± 0.40% ID/g at 60 min and 2.22 ± 0.27% ID/g at 120 min (Fig 7). Minimal radioactivity was found in the brain during this time period (Fig 6). The radioactivity in the brain was 1.16 ± 0.20% ID/g at 60 min and 0.72 ± 0.10% ID/g at 120 min (Fig 7). The tumor/brain ratios of ^18^F-tLyP-1 reached 2.69 ± 0.52 and 3.11±0.25, respectively at 1 h and 2 h post-injection and the tumor was visualized clearly (Fig 6B and 6C).

The radioactivity in the blood pool, heart, and liver was high at 30 min, but had decreased to very low levels by 60 min (Fig 6). Intense radioactivity was found in the gallbladder, intestine, kidney, and bladder from 30–120 min (Fig 6). The radioactivity levels observed in tumor were significantly higher than in the brain, lung, heart, liver, spleen, and muscle at 60 and 120 min (Fig 7; all *P<*0.001). However, the radioactivity in tumor was significantly lower than observed in the intestine at 60 and 120 min (Fig 7; all *P*<0.001). In the kidney, the level of radioactivity compared to the tumor was similar at 60 min (*P* = 0.691) and lower at 120 min (Fig 7; *P*<0.001). The biodistribution of ^18^F-tLyP-1 was similar to FAM-tLyP-1.

## Discussion

Here we show that the tLyP-1 peptide can be successfully conjugated to fluorescent and radio label markers to selectively target glioma tumors both in vitro and in vivo. The FAM-tLyP-1 reagent binds to U87MG cells at low concentrations (1μM) in vitro. In vivo, FAM-tLyP-1 accumulates in NRP-positive U87MG tumors and mirrors the distribution of NRP1. These findings suggest that FAM-tLyP-1 might be an alternative to CTX:Cy5.5. Compared with CTX, tLyP-1 is a much smaller (only 7 amino acid residues) and has a less complicated molecule structure, which will likely make it is easier to synthesize, purify, and translate to the clinic [35].

We also demonstrated that the radiolabeled ^18^F- tLyP-1 is a viable candidate for an imaging reagent. ^18^F-FDG is a commonly used PET radiopharmaceutical to detect malignancies [41–44]. However, it plays limited role in detection of malignancies in the brain because of high uptake of ^18^F-FDG in normal brain tissue [45–47]. Here we show that uptake of FAM-tLyP-1 and ^18^F- tLyP-1 in the brain was minimal. This resulted in high T/brain ratio in the brain and clear tumor visualization. Similar to ^18^F-tLyP-1, the tumor/brain ratios of FAM-tLyP-1 was also high, indicating both molecular reagents could likely by used to identify glioma microinvasions before the surgery and intraoperation to guide the resection of malignant glioma.

Given that NRPs are overexpressed in angiogenic vessels in 98–100% of carcinomas and in most malignant tumor cells [22–29], FAM and ^18^F-floride labeled tLyP-1 might have potential as broad spectrum tracers to detect other tumors. We observed relatively low levels of FAM-tLyP-1 and ^18^F-tLyP-1 uptake in the head and neck, lungs, heart, liver, spleen, bone, and muscle suggesting these reagents may be useful for detecting tumors in these regions. Unfortunately, there was high accumulation of FAM-tLyP-1 and ^18^F-tLyP-1 in the gallbladder, intestine, kidneys, and urinary bladder that would make it difficult to visualize tumors in the abdomen. This also suggested that FAM-tLyP-1 was excreted via the urinary system and hepato- biliary tract. Additional modifications of tLyP-1 are warranted to reduce the biliary excretion and improve *in vivo* pharmacokinetics.

Although we demonstrated that FAM-tLyP-1 and ^18^F-tLyP-1 can clearly visualize glioma, FAM could be replaced with near infrared fluorescent molecules (such as Cy5.5) that are more suitable for intraoperative imaging [16]. In addition, the laborious and time-consuming radiosynthesis of ^18^F-SFB requires improvement. Radiosynthesis of ^18^F-tLyP-1 using a more straightforward approach based on the chelation of aluminum fluoride by (1, 4, 7-triazacyclononane-1, 4, 7-triacetic acid) might be a more efficient alternative [48].

## Conclusion

This study demonstrated that FAM and ^18^F-labeled tLyP-1 peptide can selectively target gliomas and provide a high tumor-to-background ratio 1 h post administration. Both FAM-tLyP-1 and ^18^F-tLyP-1 are potential molecular tracers to characterize NRP receptor expression, help visualize tumors, and improve the diagnosis of glioma.

## Supporting Information

S1 Table T/NT rations of FAM-tLyp-1 and FAM-control peptide. (XLS)

S2 Table Uptake of ^18^F-tLyP-1 at 60min and 120 min after injection. (XLS)
